# Cell Signaling in Model Plants 2.0

**DOI:** 10.3390/ijms22158007

**Published:** 2021-07-27

**Authors:** Jen-Tsung Chen, Parviz Heidari

**Affiliations:** 1 Department of Life Sciences, National University of Kaohsiung, Kaohsiung 81148, Taiwan; 2 Faculty of Agriculture, Shahrood University of Technology, Shahrood 3619995161, Iran; heidar-ip@shahroodut.ac.ir

Plant cell signaling is an intensive research topic in which reductionist can be achieved when we investigate the systems of model plants [1]. To continue our previous Special Issue “Cell Signaling in Model Plants”, the second volume explores more insights into the regulatory mechanisms of plant growth and development and eventually collects 18 publications that consist of ten original research articles and eight literature reviews.

Among the model plants, *Arabidopsis thaliana* is the most utilized system for revealing the in-depth mechanisms of cell signaling in this Special Issue. Krogman et al. investigated the concentration of cytosolic calcium ion ([Ca^2+^]_cyt_) in different root cells of *A. thaliana* that express *GCaMP3* for producing a modified version of a genetically encoded calcium indicator [2]. According to the results, the authors confirmed that GCaMP3 cell lines could be a reliable system for studying the connections between an environmental stimulus and root development that communicate through calcium signaling. Mohammad-Sidik et al. studied the response and underlying mechanisms of [Ca^2+^]_cyt_ that are elevated by extracellular ATP (eATP) and ADP (eADP) in the root of *A. thaliana* [3]. The authors identified AtANN1 (annexin 1) that mediates the increase of [Ca^2+^]_cyt_ in root cells in response to both eATP and eADP, which might be a reactive oxygen species (ROS)-activated Ca^2+^ channel in the plasma membrane. The mechanism that regulates the size of *Arabidopsis* root meristem was investigated by Hashem et al. [4] with an emphasis on the involvement of hormone crosstalk and ROS. Finally, the authors suggest that polyamines modulate the size of root meristem and that it is through the interaction with auxin and cytokinin signaling and the accumulation of ROS is achieved. In the report by Villacampa et al. [5], it was demonstrated that the growth and proliferation of meristematic cells could be affected by microgravity and partial gravity together with red light photostimulation. The authors proposed that the resulting data might be valuable in the future for designing bioregenerative life support systems and space farming. The protein family of mitochondrial transcription termination factor (mTERF) has been identified as a key player in plant development, abiotic stress tolerance, etc. Jiang et al. contributed a report studying the function of *Arabidopsis* mTERF27 when faced with salt stress [6]. It was confirmed that mTERF27 interacted with multiple organellar RNA editing factor 8 (MORF8) directly, which may be crucial for regulating mitochondrial gene expression and thus affecting the development of mitochondria under salt stress. Cyclic nucleotide-gated channels (CNGCs) are a group of cation channels, and 20 subunits of CNGCs have been identified in *A. thaliana*. They have been found to participate in the development, defense, and stress response in plants. Jarratt-Barnham et al. indicated that the heterotetrameric complexes of CNGC subunits act differently from the homotetramers and that cyclic nucleotide-gated channel-like proteins ma bey involved in the regulation of the complex formation [7].

In plant research, the exploration of cell signaling pathways is crucial for breeding stress-tolerant crops. Min et al. studied the role of clade A Type 2C protein phosphatases in rice (OsPP2C09) with an emphasis on the relationship with abscisic acid (ABA) signaling under osmotic stress [8]. The authors concluded that OsPP2C09 regulates an ABA-independent signaling pathway through activating promoters that contain a drought-responsive element. Under stress conditions, lipid second messengers including phosphatidic acid, phosphoinositide, lysophospholipids, and sphingolipids, could modulate the physiological responses of plants. Rodas-Junco et al. summarized the involvement of lipid second messengers in plant cell signaling under osmotic stress and the knowledge is critical for the development of strategies to generate stress-tolerant crops [9]. Lyu et al. investigated the roles of the gene family, *CitGF14s*, that codes for phosphorylated proteins in *Citrus sinensis* in response to biotic and abiotic stresses [10]. The authors found certain isoforms of CitGF14s displayed tissue-specific expression patterns and unraveled the protein–protein interaction network. Finally, they confirmed that several candidates may play an important role in regulating the development and stress responses in *C. sinensis*. A receptor for brassinosteroid signaling, SlBRI1, was modified in a phosphorylation site of serine 1040 and then tested for its functions in tomatoes [11]. Wang et al. found that the modified SlBRI1 exhibited a positive response in term of both growth and yield under heat stress. It was thus confirmed that the phosphorylation site of ser-1040 in SlBRI1 affects heat tolerance and might be applied in protecting growth and yield from high-temperature stress in crops. In response to biotic and abiotic stresses, a significant reprogramming was found to occur in the level of plant transcriptome. Chong et al. contributed a review article for refining the knowledge of signal integration by mediators, particularly the cyclin-dependent kinase 8 (CDK8) module [12]. The CDK8 module was thought to predominantly act as a transcriptional repressor, however, it may play a contrasting regulatory role that depends on the type of biotic and abiotic stress.

In cell signaling, the knowledge obtained from model plants could be applied in crops to accelerate the progress of research and breeding programs. FLAVIN-BINDING, KELCH REPEAT, F-BOX 1 (FKF1) has been recognized as a blue-light receptor that plays a role in the promotion of flowering in *A. thaliana*. Shibuya et al. identified an FKF1 homolog SlFKF1 in tomatoes and confirmed that the expression of *SlFKF1* at a low level was associated with late flowering, increased leaflets, and low concentrations of lycopene [13]. A mitogen-activated protein kinase (MAPK) is a type of protein kinase that has been well-proved in *A. thaliana* to play a role in defense responses. Additionally, a MAPK in *Lotus japonicus*, LjMPK6, was known to be involved in regulating symbiosis between legume and rhizobia. Yan et al. identified a type 2C protein phosphatase, LjPP2C, and confirmed that it is required for dephosphorylating LjMPK6 to regulate the nodule development in *L. japonicas* [14]. Chaulagain and Frugoli contributed a review article on the advances in the symbiosis between legumes and rhizobia with an emphasis on the regulation of nodule number [15]. The signaling pathways for initiation and organogenesis of the nodule, nitrate-dependent signaling, and autoregulation of nodulation were comprehensively discussed, and it set up the direction of future research on the fine-tuning of the plant’s response to rhizobia.

Magnesium ions (Mg^2+^) are the second most abundant cation in living cells and they serve as a signal of the adenylate status. Kleczkowski and Igamberdiev refined the signaling pathway of Mg^2+^, particularly the adenylate kinase (AK) equilibrium and its involvement in the adenylate status [16]. It was concluded that AK plays a central role in Mg^2+^ signaling and is an allosteric effector in cellular metabolism for energy homeostasis in cells.

ROS are a universal regulatory element in plant cells and participate in an array of signaling pathways. Breygina and Klimenko reviewed the role of ROS and the underlying mechanism involving ion transport systems during plant sexual reproduction [17]. They found that ROS are involved at most stages of the life cycle in the male gametophyte, and it chiefly acts through ion currents by a feedback loop.

Parasitic plants absorb water and nutrients from hosts and could cause substantial losses of yield. Hu et al. summarized the virulence mechanisms in obligate root parasites of the genera *Orobanche* and *Striga* focusing on the activities of proteins with nucleotide-binding and leucine-rich repeat domains, NLR proteins, that encoded by resistance genes of the host [18]. This present review presents recent advances in this field achieved by CRISPR genome editing and RNAi silencing and indicates that the new findings might contribute to establishing strategies for controlling parasitic weeds.

The communication between the nucleus, organelles, and cellular compartments is a critical system for plant cells to face changing environments, including stresses. Mielecki et al. contributed a review for a better understanding of retrograde signaling in stress responses, such as its involvement in the induction of cell death and in the biogenesis of organelles [19]. The authors stated that the knowledge may be important for developing novel strategies for improving the adaptability of plants in changing environments.

Theoretically, cell signaling affects virtually every aspect of plant cell structure and function, and thus to scientists, unveiling the global network and the underlying machinery is always a great challenge. This Special Issue only presents a snapshot of this intensive field of plant biology and we believe that with the rapid development of advanced technology such as integrative multi-omics and CRISPR genome editing, more and more critical networks of plant cell signaling will be unraveled in the near future.
